# Influence of Surgeon Experience on Surgical Outcome of Maxillomandibular Advancement for Obstructive Sleep Apnea

**DOI:** 10.3390/jcm12103504

**Published:** 2023-05-16

**Authors:** Jean-Pierre T. F. Ho, Semih Özkan, Ning Zhou, Ruben C. Apperloo, Naichuan Su, Alfred G. Becking, Jan de Lange

**Affiliations:** 1 Department of Oral and Maxillofacial Surgery, Amsterdam UMC, University of Amsterdam, Meibergdreef 9, 1105 AZ Amsterdam, The Netherlands; 2 Academic Centre for Dentistry Amsterdam (ACTA), University of Amsterdam and Vrije Universiteit Amsterdam, 1081 LA Amsterdam, The Netherlands; 3 Department of Oral and Maxillofacial Surgery, Northwest Clinics, 1815 JD Alkmaar, The Netherlands; 4 Department of Orofacial Pain and Dysfunction, Academic Centre for Dentistry Amsterdam (ACTA), University of Amsterdam and Vrije Universiteit Amsterdam, 1081 LA Amsterdam, The Netherlands; 5 Department of Oral and Maxillofacial Surgery, St Antonius Hospital, 3435 CM Nieuwegein, The Netherlands; 6 Department of Oral Public Health, Academic Center for Dentistry Amsterdam (ACTA), University of Amsterdam and Vrije Universiteit Amsterdam, 1081 LA Amsterdam, The Netherlands

**Keywords:** maxillomandibular advancement, obstructive sleep apnea, postoperative complications, surgical cure, surgical success, surgeon experience

## Abstract

The primary aim of this study was to assess the association between clinical efficacy outcomes (i.e., polysomnography (PSG) results) of maxillomandibular advancement (MMA) and surgeons’ experience. The second aim was to assess the association between the occurrence of postoperative complications of MMA and surgeons’ experiences. Patients treated with MMA for moderate to severe obstructive sleep apnea (OSA) were enrolled in this retrospective study. The patient population was divided into two groups based on two different surgeons performing MMA. The associations between surgeons’ experience on the one hand and PSG results and postoperative complications on the other hand were investigated. A total of 75 patients were included. There was no significant difference in baseline characteristics between the two groups. The reductions in apnea-hypopnea index and oxygen desaturation index were both significantly greater in group-B than group-A (*p* = 0.015 and 0.002, respectively). The overall success rate after MMA was 64.0%. There was a negative correlation between surgeon experience and surgical success (odds ratio: 0.963 [0.93, 1.00], *p* = 0.031). No significant association was found between surgeon experience and surgical cure. Additionally, there was no significant association between surgeon experience and the occurrence of postoperative complications. Within the limitations of this study, it is concluded that surgeon experience may have little to no influence on the clinical efficacy and safety of MMA surgery in OSA patients.

## 1. Introduction

Obstructive sleep apnea (OSA) is a sleep breathing disorder where patients have repetitive episodes of partial or complete upper airway collapse and obstruction during sleep. This leads to absent and/or reduced respirations during sleep, which are called apneas and hypopneas, respectively [1]. It is estimated that globally, approximately 425 million adults between the ages of 30 and 69 have moderate to severe OSA [2]. OSA has been linked as an independent risk factor for cardiovascular, cerebrovascular, and metabolic diseases, reduced neurocognitive function, and increased mortality [3,4,5,6,7,8].

Since the inception of continuous positive airway pressure (CPAP) in 1981, it is currently still considered the first-line treatment of choice for moderate to severe OSA [9,10,11]. Nevertheless, patients and physicians may choose an alternative to CPAP due to, e.g., poor adherence to CPAP and the desire for a more tailor-made treatment based on patient preference, patient-reported outcome parameters, individual risk, and patient OSA phenotype [12,13,14].

Alternatives to CPAP therapy mainly include behavior strategies (e.g., weight loss), medical therapy (e.g., mandibular advancement devices (MADs)), and surgical therapy (e.g., upper airway surgery) [15]. Of the non-surgical alternative options to CPAP for the treatment of OSA, MADs are the most common modality. MADs advance the mandible in order to increase the airway volume and decrease the pharyngeal collapsibility [16]. There are different MAD designs, but a clear clinically relevant distinction in favor of one of the appliances cannot be drawn at this stage [16,17,18,19]. Given the variable efficacy and nature of life-long treatment with MADs, combined with potential side effects such as unwanted dental and skeletal changes, acceptance and adherence to MADs may decrease in OSA patients [17,20].

A surgical alternative with similar success rates to CPAP is maxillomandibular advancement (MMA) [14,21,22]. MMA is a skeletal surgery that addresses the entire upper airway [10]. It consists of surgical advancement of the maxilla and mandible—often combined with counterclockwise rotation of the maxillomandibular complex—by performing a combination of a Le Fort I osteotomy of the maxilla and a bilateral sagittal split osteotomy of the mandible [14,23]. Although there is only limited evidence on the association between the magnitude of advancement and reduction in AHI following MMA, a mandibular advancement of at least 10 mm has been recommended in MMA surgery for OSA [24,25]. By displacing the facial bones, MMA is able to enlarge multiple levels of the upper airway in both the medio-lateral and antero-posterior dimensions. In addition to enlarging the upper airway, MMA also increases tension and decreases collapsibility of the suprahyoid and velopharyngeal muscles [26,27].

Different aspects of MMA have been investigated in the past in order to see whether they are associated with a higher MMA success rate, e.g., certain patient characteristics, specific comorbidities, particular polysomnography parameters, specific drug-induced sleep endoscopy findings, and certain surgical aspects [28,29,30,31,32,33,34].

However, one aspect that has not been investigated is whether MMA-related surgical experience has any bearing on the outcome after MMA. In many fields—such as general surgery or orthopedic surgery—it has been proven that operative results and health-related quality of life following surgery were significantly and positively correlated with surgeon experience and that outcomes of patients treated by less experienced surgeons were slightly worse than those treated by more experienced surgeons [35,36,37,38].

The aims of this study were (1) to assess the association between clinical efficacy outcomes (i.e., polysomnography (PSG) results) of MMA and surgeons’ experience; and (2) to assess the association between the occurrence of postoperative complications of MMA and surgeons’ experience. The hypothesis is that surgical outcomes after MMA are better when surgeons’ MMA-related surgical experience increases.

## 2. Materials and Methods

### 2.1. Ethical Considerations

This study was deemed not to be subject to the Medical Research Human Subjects Act by the Medical Ethics Committee of the Amsterdam University Medical Centers (UMC), location Academic Medical Center (AMC) (reference number W23_017 # 23.041). A formal approval was therefore waived. Patients were sent a letter to inform them that their medical records, radiological images, and test results were going to be used for study purposes. They were given the option to object and opt out of inclusion in the study. This study was performed in accordance with the Declaration of Helsinki guidelines for human research, 1964, as amended in 2013 (64th WMA General Assembly, Fortaleza, Brazil). It was conducted at the Department of Oral and Maxillofacial Surgery of the Amsterdam UMC, The Netherlands.

### 2.2. Study Participants

We performed a single-center retrospective study including a consecutive series of patients with OSA undergoing MMA surgery between January 2012 and March 2021 at the Department of Oral and Maxillofacial Surgery at the Amsterdam UMC, location AMC. Patients who met the inclusion criteria were eligible for this study.

The inclusion criteria were: (1) adults aged ≥18 years; (2) diagnosis of moderate to severe OSA (apnea-hypopnea index (AHI) ≥15 events/h) as determined by a preoperative overnight polysomnography (PSG); (3) continuous positive airway pressure (CPAP) therapy failure or intolerance; and (4) presence of a follow-up PSG at least 3 months postoperatively. The exclusion criteria were: (1) no consent to the use of the patient record data for research purposes; (2) patients who underwent other adjunctive procedures at the time of MMA (e.g., multi-piece Le Fort osteotomy, temporomandibular joint reconstruction); (3) previous history of Le Fort I osteotomy or bilateral sagittal split osteotomy (BSSO); and (4) cleft palate and/or craniofacial syndromic patients.

The included medical records were reviewed, and data was collected. Preoperative (baseline) patient data included gender, age, and body mass index (BMI). The included patients were divided into two groups based on two different oral and maxillofacial surgeons (RA and JH), who performed the MMA surgery. Patients who were operated on by surgeon A were designated as group-A; patients who were operated on by surgeon B were designated as group-B.

### 2.3. Surgeon Experience

Surgeon A started performing orthognathic and MMA surgery in March 2012; surgeon B first started performing orthognathic and MMA surgery in June 2017. For both surgeons, surgeon experience—i.e., MMA surgery-related experience—was calculated (in months) by subtracting the surgeon’s starting date from the date on which MMA surgery was performed for each patient.

### 2.4. Maxillomandibular Advancement Surgery

All MMA procedures were completed using standardized surgical techniques by the two surgeons, which included a Le Fort I osteotomy for the maxilla in combination with a Hunsuck-Dal Pont modification of the Obwegeser BSSO for the mandible. Both the maxilla and mandible were advanced anteriorly and, whenever possible, counter-clockwise rotated [23].

Prior to the availability of three-dimensional (3D) planning, patients were treated with a traditional two-dimensional (2D) planned surgical procedure using a standard surgical protocol with the goal of 8–10 mm advancement. Manually manufactured intraoperative occlusal splints were utilized in the planned 2D surgical procedure. After the availability of 3D planning, patients were virtually planned, and the degree of advancement was personalized based on multiple patient-related factors, including severity of OSA, skeletal pattern, dental occlusion, facial characteristics, prior upper airway surgery, and collapse pattern of the upper airway when pre-MMA drug-induced sleep endoscopy was available. Computer-aided design/computer-aided manufacturing of intraoperative occlusal splints were used in the 3D-planned surgical procedure [14].

Immediately postoperatively, all patients received extensive postoperative monitoring in either the intensive or medium care unit [39,40]. After being discharged from the intensive or medium care unit, the patients were transferred to a general post-surgery ward for further recovery [40].

### 2.5. Polysomnography

All patients underwent a level 1 or level 2 PSG preoperatively and at least 3 months postoperatively. PSG recordings were manually checked and scored according to the standards of the American Academy of Sleep Medicine (AASM) manual for the scoring of sleep and associated events [41]. The collected preoperative and postoperative PSG variables included AHI, oxygen desaturation index (ODI), and lowest oxyhemoglobin saturation (LSAT).

Based on Sher’s criteria, surgical success was defined as an AHI reduction of at least 50% and an AHI below 20 events/h postoperatively [42]. Surgical cure was defined as a postoperative AHI below 5 events/h [43].

### 2.6. Postoperative Complication

Postoperative complications related to MMA surgery were assessed during the follow-up for each patient. These were classified as minor or major complications according to the criteria of the “Accordion severity classification of postoperative complications” by Strasberg et al. [44].

### 2.7. Statistical Analysis

All data were analyzed using SPSS software (version 27, IBM Corp., Armonk, NY, USA). A descriptive statistical analysis was performed for all demographic and outcome variables. Continuous variables were presented as mean and standard deviation (SD), and categorical variables were reported as frequency and percentage.

In order to compare baseline characteristics and surgical variables between group-A and group-B, the independent samples *t*-test was used. To determine how PSG values change from pre- to post-operative time between groups A and B, a two-way ANOVA test with one factor repeated was used. To investigate the association of surgeon experience with surgical success or cure, multivariate binary logistic regression analyses were used, with surgical success or surgical cure as dependent variables and surgeon experience as an independent variable, adjusted for surgeon groups (A and B), age, gender, baseline BMI, and baseline AHI. To investigate the association between surgeon experience and the AHI reduction after MMA, multivariate linear regression was used, with AHI reduction as the dependent variable and surgeon experience as the independent variable, adjusted for surgeon groups (A and B), age, gender, baseline BMI, and baseline AHI. To analyze the correlation between surgeon experience and the occurrence of postoperative complications, multivariate ordinal regression was used with postoperative complications as the dependent variable and surgeon experience as the independent variable, adjusted for surgeon groups (A and B), age, gender, smoking, degree of mandibular advancement, degree of maxillary advancement, baseline BMI, and baseline AHI.

## 3. Results

At the department of Oral and Maxillofacial Surgery at the Amsterdam UMC, location AMC, a total of 80 patients underwent MMA for moderate to severe OSA either by surgeon A or surgeon B. Among these patients, two declined for their patient data to be used for research purposes; one was excluded due to the absence of available preoperative PSG data; and two were excluded because they underwent temporomandibular joint reconstruction in conjunction with MMA. Therefore, 75 patients were included in this study.

### 3.1. Baseline Characteristics of Group-A versus Group-B

Group-A and group-B, consisted of 49 (65.3%) and 26 (34.7%) patients, respectively. In total, there were 64 males (85.3%) and 11 females (14.7%). The mean age was 50.7 ± 10.0 years, with a mean BMI of 30.2 ± 4.2 kg/m^2^ for the total study population. There were 72 patients (96.0%) who presented with CPAP intolerance or failure prior to MMA. Additionally, 32 patients (42.6%) received a form of upper airway surgery for OSA prior to MMA. The mean preoperative AHI was 54.8 ± 21.3 events/h. There was no significant difference found between group-A and group-B in baseline characteristics. Baseline demographic characteristics and PSG values of the total population, group-A, and group-B are presented in Table 1.

### 3.2. Surgical Characteristics of Group-A versus Group-B

When comparing surgical variables between group-A and group-B, it was found that the degree of maxillary advancement, the degree of mandibular advancement, and the total operation time did not significantly differ between the 2 groups (*p* = 0.260; *p* = 0.078; *p* = 0.051, respectively). Anticlockwise rotation of the maxillomandibular complex was performed in 20 patients in group-A (52.6%) and in 24 patients in group-B (96.0%). This difference between the two groups was, however, found to be statistically significant (*p* < 0.001). The mean blood loss during surgery in group-A was significantly lower than that in group-B (347.6 ± 193.3 cubic centimeters [cc] vs. 455.6 ± 268.5 cc, *p* < 0.001) (Table 2).

### 3.3. Postoperative Outcomes of Group-A versus Group-B

The preoperative and postoperative PSG values are shown in Table 3. AHI, ODI, and LSAT were all significantly improved for both group-A and group-B (*p* < 0.001) after MMA. In group-A the mean AHI decreased from 54.0 ± 21.6 to 20.0 ± 17.4 events/h, compared to a decrease from 56.8 ± 21.2 to 14.9 ± 15.7 events/h in group B, respectively. The improvement in AHI in group-B was significantly larger compared to that in group-A (*p* = 0.015). The mean ODI decreased from 50.1 ± 20.9 to 28.7 ± 18.4 events/h in group-A, compared to a decrease from 62.3 ± 21.3 to 17.8 ± 11.8 events/h in group-B. The reduction of ODI in group-B was significantly larger than that in group-A (*p* = 0.002). In contrast to the AHI and ODI, no significant difference was found between the two groups for the improvement of the LSAT after MMA (*p* = 0.163).

### 3.4. Correlation between Surgeon Experience and Surgical Outcome

In the total study population, surgical success was achieved in 48 patients (*n* = 48/75; 64.0%). Surgical success was achieved in 29 patients (*n* = 29/49; 59.2%) and in 19 patients (*n* = 19/26; 76.0%), in group-A and group-B respectively. Surgical cure was achieved in 17 patients (*n* = 17/75; 22.7%) in the total population, 12 patients (*n* = 12/49; 24.5%) in group-A and 5 patients (*n* = 5/26; 20.0%) in group B. The mean surgical experience of surgeon A was 34.0 ± 20.7 months, and the mean surgical experience of surgeon B was 23.4 ± 11.0 months. There was no significant difference found between the two-groups in surgical success (*p* = 0.065) or surgical cure (*p* = 0.151). The results of the binary logistic regression analyses—in order to investigate the correlation between surgeon experience and surgical cure or surgical success, adjusted for surgeon group (A and B), age, gender, baseline BMI, and baseline AHI—are shown in Table 4. There was a slightly negative correlation between surgeon experience and surgical success (odds ratio: 0.963 [0.93, 1.00], *p* = 0.031). No significant correlation was found between surgeon experience and surgical cure (*p* = 0.535).

### 3.5. Correlation between Surgeon Experience and AHI Reduction

The results of the linear regression—in order to investigate the correlation between surgeon experience and AHI reduction—are shown in Table 5. There was no significant correlation found between surgeon experience and AHI reduction (*p* = 0.489).

### 3.6. Correlation between Surgeon Experience and Occurrence of Postoperative Complications

Twenty-four of 75 patients (32%; 19 in group A and 5 in group B) did not experience any postoperative complications. Minor complications occurred in 25 patients (*n* = 25/75; 33.3%) of the total study population; 11 patients (*n* = 11/49; 22.4%) in group-A; and 14 patients (*n* = 14/26; 53.8%) in group-B. Major complications occurred in 26 patients (*n* = 26/75; 34.7%) of the total study population; 19 patients (*n* = 19/49; 38.8%) in group-A; and 7 patients (*n* = 7/26; 26.9%) in group-B (Table 6). The results of the correlation between surgeon experience and the occurrence of postoperative complications are shown in Table 7. There was no significant correlation found between surgeon experience and the occurrence of postoperative complications (*p* = 0.656).

## 4. Discussion

This study was set out with the aim of assessing whether surgeon experience influences the surgical outcomes of MMA. In order to do so, clinical efficacy outcomes (i.e., polysomnography (PSG) results) and postoperative complications of MMA surgeries—performed by two oral and maxillofacial surgeons with different surgical experiences—were analyzed. As far as the authors are aware, this is the first study that has specifically looked into these issues related to MMA outcomes.

The study results show that, in contrast to general and orthopedic surgery literature, which has shown that surgeon experience is correlated with surgical outcomes, surgeon experience was slightly negatively associated with surgical success and was not associated with surgical cure [35,36,37,38]. To further investigate this finding, we investigated whether surgeon experience has any correlation with AHI reduction to complement the effect of surgeon experience on MMA. This, however, was also found not to be the case. This might be explained by the fact that more experienced surgeons might have strived to treat the more complicated OSA cases. A different possible explanation for these results might be the inherent complexity of OSA itself. Other authors have previously stated that in addition to anatomical factors, non-anatomical factors—e.g., critical closing pressure, loop gain, muscle responsiveness, and arousal threshold—might play an important role in OSA phenotyping and the treatment outcome of the OSA patient [31,34,45,46,47,48,49]. These features are, of course, independent of surgeon experience and could therefore explain these findings.

Although no correlation was found between surgeon groups (A and B) and surgical outcomes, when evaluating the preoperative and postoperative PSG values between the two-groups, it was found that the decreases in AHI and ODI were significantly greater in group-B compared to group-A. A possible explanation for this might be that the preoperative AHI and ODI in group-B were slightly higher compared to group-A. As a result, a greater reduction in AHI and ODI following MMA was possible. Another explanation could be the difference in surgical techniques between the two surgeons. As the use of anticlockwise rotation of the jaw was significantly higher and the degree of mandibular advancement tended to be larger in group-B, this may have contributed to a more favorable effect on the upper airway [23]. Hence, sufficient jaw advancement may be an important factor associated with MMA treatment outcome, which needs to be further investigated in future research.

Additionally, the results showed that surgeon experience was not associated with the occurrence of postoperative complications after MMA. Similar to the reported literature, this study’s results illustrate that plate infection and removal, which is considered a major complication, is the main indication requiring readmission and/or reoperation [21,44]. When looking at the minor complications, these study results also show that neurosensory disturbance proved to be the most reported (minor) complication after MMA [21,22].

When interpreting the results of this study, one should bear in mind that there are several factors that must be taken into consideration in addition to certain limitations of this study. Due to the retrospective study design, there is, of course, a possibility for selection bias [50]. Secondly, the sample size of the two-groups could be considered quite small, which could therefore potentially lead to sampling bias [51]. When looking at the size of the two-groups, there could be a potential for selection bias [50]. However, when looking at the baseline characteristics for the two-groups, no significant difference was found, and these could be regarded as homogenous. Thirdly, the same previous argument can be made for the limited number of surgeons (*n* = 2) evaluated in this study. Fourthly, the overall success rate in this study is lower than reported elsewhere in the literature [21,25]. This could be due to the fact that MMA in our center is mainly indicated for more severe to extreme OSA patients (mean AHI of 54.8 events/h), whereas other studies also include more moderate to less severe OSA patients [52,53]. In addition to a higher preoperative AHI, the average age among patients included in this study was also higher compared to other studies on the surgical outcome of MMA [21]. These findings could potentially not be extrapolated to all patients and therefore be limited to this specific patient profile. Fourth, PSG variables and complications might be insufficient to address our research question. Finally, comparable to earlier papers, this study defined surgeon experience as the number of months of practice [35,38,54]. Some might argue that experience might be better measured in case volume [37,38]. When looking at the number of cases between the two-groups, surgeon A almost had twice as many cases as surgeon B; nevertheless, the study results exhibited that this difference in volume between the two surgeons proved to have no bearing on surgical outcome or the occurrence of complications between the two surgeons. It should be noted that many factors are involved in a surgeon’s experience, such as the number of surgeries he/she performed during training, additional training—for example, fellowships—and the experience level of the training surgeon’s tutor, which are not taken into account in this study.

In order to better investigate the association between the surgeon’s experience and MMA surgical outcome, as part of the standards of MMA surgery care for patients with OSA, the authors do recommend that prospective studies with a large sample size be executed. In addition, more research is necessary on different aspects of MMA, such as surgical techniques, quality of life, and long-term outcome [55,56,57]. In addition to MMA, other alternatives to CPAP, such as MADs and hypoglossal nerve stimulation, have shown promising outcomes in well-selected OSA patients [15]. Therefore, for OSA patients who refuse CPAP therapy, alternative treatments should be considered based on both patient phenotypes and patient preferences.

In spite of the limitations of this study and the fact that no association was found between surgeon experience and surgical outcome, the authors still feel that the study is relevant and provides insight into factors that might and might not notably contribute to the surgical outcome after MMA.

## 5. Conclusions

Within the confines of the study limitations, the findings reject the hypothesis that surgical outcome after MMA is better—with regards to surgical success, surgical cure, and lower occurrence of complications—when the surgeons’ experience increases.

## Figures and Tables

**Table 1:** Baseline characteristics of the total population, group-A and group-B.

	Total Population (*n* = 75)	Group-A (*n* = 49)	Group-B (*n* = 26)	*p*-Value
Male:female (*n*)	64:11	43:6	21:5	0.423
Age (years)	50.7 ± 10.0	50.7 ± 9.5	50.8 ± 11.0	0.969
BMI (kg/m^2^)	30.2 ± 4.2	30.6 ± 4.4	29.5 ± 3.6	0.307
AHI (events/h)	54.8 ± 21.3	54.0 ± 21.6	56.2 ± 21.0	0.676
ODI (events/h)	54.8 ± 21.7	50.1 ± 20.9	62.3 ± 21.3	0.073
LSAT (%)	76.1 ± 11.0	76.6 ± 11.3	75.3 ± 10.6	0.634

Data presented as mean ± standard deviation. *p*-values comparing group-A and group-B. *p*-value < 0.05 is considered statistically significant. AHI, apnea hypopnea index; BMI, body mass index; LSAT, lowest oxyhemoglobin saturation; ODI, oxygen desaturation index.

**Table 2:** Surgical characteristics of the total population, group-A and group-B.

	Total Population (*n* = 75)	Group-A (*n* = 49)	Group-B (*n* = 26)	*p*-Value
Maxillary advancement (mm)	7.1 ± 2.4	7.4 ± 2.7	6.7 ± 1.9	0.260
Mandibular advancement (mm)	9.7 ± 4.4	8.9 ± 4.4	10.9 ± 4.1	0.078
Anticlockwise rotation of the jaw (%)	69.8	52.6	96.0	<0.001
Operation time (min)	222.2 ± 60.3	205.5 ± 60.1	253.8 ± 47.4	0.051
Blood loss (cc)	384.1 ± 225.6	347.6 ± 193.3	455.6 ± 268.5	<0.001

Data of maxillary advancement, mandibular advancement, operation time, and blood loss are presented as mean ± standard deviation. Data of rotation is presented as percentage. *p*-values compare surgical variables between group-A and group-B. *p*-value < 0.05 considered statistically significant.

**Table 3:** Preoperative and postoperative polysomnography values for group-A and group-B.

		Preoperative	Postoperative	*p*-Value	Δ	*p*-Value *
AHI (events/h)	Group A Group B	54.0 ± 21.6 56.8 ± 21.2	20.0 ± 17.4 14.9 ± 15.7	<0.001 <0.001	34.0 ± 23.2 41.9 ± 24.5	0.015
ODI (events/h)	Group A Group B	50.1 ± 20.9 62.3 ± 21.3	28.7 ± 18.4 17.8 ± 11.8	<0.001 <0.001	21.4 ± 20.0 44.5 ± 25.1	0.002
LSAT (%)	Group A Group B	76.7 ± 11.6 75.0 ± 10.9	85.1 ± 5.9 84.0 ± 7.3	<0.001 <0.001	8.3 ± 11.0 9.0 ± 8.4	0.163
Success (*n*, (%))	Group A Group B	-	29 (59.2) 19 (73.1)	-	-	0.065
Cure (*n*, (%))	Group A Group B	-	12 (24.5) 5 (19.2)	-	-	0.151

Data presented as mean ± standard deviation. *p*-values compare preoperative and postoperative polysomnography values. *p*-value * compare Δ (preoperative and postoperative change) between group-A and group-B. *p*-value < 0.05 is considered statistically significant. AHI, apnea hypopnea index; LSAT, lowest oxyhemoglobin saturation; ODI, oxygen desaturation index.

**Table 4:** Results of binary logistic regression for surgeon experience and surgical success and cure.

Variable	B	S.E.	Exp(B)	95% CI	*p*-Value
Surgical Success					
Constant	6.958	3.527	1051.166	-	0.049
Surgeon experience (month)	−0.037	0.017	0.963	[0.931, 0.997]	0.031
Age (years)	−0.082	0.034	0.921	[0.863, 0.984]	0.015
Gender					
Female (Ref.)					
Male	−0.768	0.806	0.464	[0.095, 2.253]	0.341
Baseline BMI (kg/m^2^)	−0.022	0.074	0.978	[0.847, 1.130]	0.766
Baseline AHI (events/h)	0.004	0.013	1.004	[0.979, 1.030]	0.750
Surgeon	0.407	0.623	1.502	[0.443, 5.097]	0.514
**Surgical cure**					
Constant	0.717	3.417	2.049	-	0.834
Surgeon experience (month)	−0.012	0.019	0.989	[0.953, 1.025]	0.535
Age (years)	−0.038	0.033	0.962	[0.902, 1.026]	0.241
Gender Female (Ref.) Male	−1.077	0.867	0.340	[0.062, 1.863]	0.214
Baseline BMI (kg/m^2^)	0.051	0.072	1.053	[0.914, 1.212]	0.476
Baseline AHI (events/h)	−0.004	0.014	0.996	[0.970, 1.024]	0.791
Surgeon group	−0.637	0.690	0.529	[0.137, 2.046]	0.356

The results are adjusted for age, gender, baseline BMI, and baseline AHI. *p*-value < 0.05 is considered statistically significant. AHI, apnea hypopnea index; BMI, body mass index; CI, confidence interval for B; SE, standard error. Adjusted for age, gender, baseline BMI, and baseline AHI.

**Table 5:** Results of linear regression for surgeon experience and the AHI reduction after MMA.

Variable	B	S.E.	95% CI	*p*-Value
AHI Reduction				
Constant	15.745	20.319	[−24.812, 56.303]	0.441
Surgeon experience (month)	−0.074	0.106	[−0.286, 0.138]	0.489
Age (years)	−0.417	0.186	[−0.787, −0.046]	0.028
Gender Female (Ref.) Male	−2.750	5.312	[−13.354, 7.854]	0.606
Baseline BMI (kg/m^2^)	−0.035	0.451	[−0.935, 0.865]	0.938
Baseline AHI (events/h)	0.845	0.084	[0.678, 1.013]	<0.001
Surgeon group	4.527	3.924	[−3.305, 12.359]	0.253

The results are adjusted for age, gender, baseline BMI, and baseline AHI. *p*-value < 0.05 considered statistically significant. AHI, apnea hypopnea index; BMI, body mass index; CI, confidence interval for B; SE, standard error.

**Table 6:** Occurrence of postoperative complications for the total population, group-A and group-B.

Complications	Number of Events (% of Population)	Group-A	Group-B
Minor complication - Neurosensory disturbance	38 (50.7)	18 (24.0)	20 (26.7)
Major complication - Osteosynthesis infection - Malocclusion - Non-union	17 (22.7) 5 (6.7) 4 (5.3)	11 (14.7) 5 (6.7) 3 (4.0)	6 (8.0) 0 (0) 1 (1.3)
**Complications**	**Number of Subjects** **(% of Population)**	**Group-A**	**Group-B**
No complication	24 (32.0)	19 (25.3)	5 (6.7)
Any complication - Minor complication - Major complication	25 (33.3) 26 (34.7)	11 (14.7) 19 (25.3)	14 (18.7) 7 (9.3)

Complications are categorized as major and minor complications. Complications are presented as number of events and number of patients for the total population, group-A, and group-B.

**Table 7:** Results of ordinal regression for surgeon experience and occurrence of postoperative complications.

Variable	B	S.E.	Exp(B)	95% CI	*p*-Value
Constant for Minor complications Constant for Major complications	−1.274 0.307	2.809 2.806	0.280 1.359	[0.001, 68.786] [0.006, 332.620]	0.650 0.913
Surgeon Experience (months)	−0.008	0.017	0.992	[0.960, 1.026]	0.656
Age (years)	0.026	0.027	1.026	[0.974, 1.081]	0.333
Gender Female Male (Ref.)	−0.957	0.835	0.384	[0.075, 1.974]	0.252
Baseline BMI (kg/m^2^)	−0.018	0.064	0.982	[0.866, 1.114]	0.776
Baseline AHI (events/h)	−0.005	0.013	0.995	[0.969, 1.022]	0.734
Mandibular advancement (mm)	0.139	0.094	1.149	[0.956, 1.381]	0.139
Maxillary advancement (mm)	−0.059	0.188	0.943	[0.652, 1.362]	0.753
Smoking (no smoking) Smoking (<10 p/week) Smoking (>10 p/week) (Ref.)	−1.672 −1.504	0.845 0.961	0.188 0.222	[0.036, 0.984] [0.034, 1.462]	0.048 0.118
Surgeon group	0.108	0.68	1.114	[0.293, 4.233]	0.874

The results are adjusted for age, gender, smoking, baseline BMI, baseline AHI, mandibular advancement, and maxillary advancement. *p*-value < 0.05 is considered statistically significant. AHI, apnea hypopnea index; BMI, body mass index; CI, confidence interval for B; SE, standard error.

## Data Availability

The data presented in this study are available on request from the corresponding author.
